# Social Comparison Manifests in Event-related Potentials

**DOI:** 10.1038/srep12127

**Published:** 2015-07-17

**Authors:** Yi Luo, Chunliang Feng, Tingting Wu, Lucas S. Broster, Huajian Cai, Ruolei Gu, Yue-jia Luo

**Affiliations:** 1 State Key Laboratory of Cognitive Neuroscience and Learning, Beijing Normal University, Beijing, 100875, China; 2 Institute of Affective and Social Neuroscience, Shenzhen University, Shenzhen, 518060, China; 3 Department of Psychology, Queens College, The City University of New York, Queens, NY 11367, USA; 4 Department of Behavioral Science, University of Kentucky College of Medicine, Lexington, KY 40506, USA; 5 Key Laboratory of Behavioral Science, Institute of Psychology, Chinese Academy of Sciences, Beijing, 100101, China

## Abstract

Social comparison, a widespread phenomenon in human society, has been found to affect outcome evaluation. The need to belong to a social group may result in distinct neural responses to diverse social comparison outcomes. To extend previous studies by examining how social comparison with hierarchical characteristics is temporally processed, electroencephalography responses were recorded in the current study. Participants played a lottery game with two pseudo-players simultaneously and received both their own and the other two players’ outcomes. Results of three event-related potential components, including the P2, the feedback-related negativity (FRN), and the late positive component (LPC), indicate that social comparison manifests in three stages. First, outcomes indicating a different performance from others elicited a larger P2 than evenness. Second, the FRN showed hierarchical sensitivity to social comparison outcomes. This effect manifested asymmetrically. Finally, large difference between the participant’s outcome and the other two players’ evoked a larger LPC than the medium difference and the even condition. We suggest that during social comparison, people detect if there is any difference between self and others, and then evaluate the information of this difference hierarchically, and finally interpret the situations in which oneself deviates from the group as most motivationally salient.

People choose and learn in social circumstances and their decisions are influenced by social contexts^1^,^2^,^3^,^4^,^5^. This kind of influence is based on social comparison between self and others^6^. That is, individuals evaluate their performances and earnings according to the comparison with those of others rather than the objective values per se. The sensitivity to social comparison facilitates the building of healthy group relations, which benefits individual and group development^7^,^8^. As a result, social comparison plays a crucial role in human survival and well-being.

With the rising of social neuroscience in recent years, an increasing number of studies have concentrated on neural underpinnings of social comparison^9^,^10^,^11^. For example, Fliessbach and colleagues found that the activation of the ventral striatum, a reward-related brain region, was affected by variations of social comparison contexts defined by the difference of payoffs between two players who performed the task simultaneously^6^. This finding was supported by following neuroimaging findings that relative reward in social comparison contexts elicits neural responses which show similar patterns with the absolute reward condition^12^,^13^. Social emotions like schadenfreude and gloating are found to be provoked by positive outcomes in social comparison, while negative outcomes in similar circumstances provoke emotions like envy and jealousy^11^,^14^. Electroencephalography (EEG) studies on social comparison have described temporal characteristics of outcome evaluation in social contexts, but their findings are heterogeneous. An event-related potential (ERP) study replicating the paradigm of Fliessbach *et al.*^6^ showed larger negative waves elicited by inequitable outcomes than equitable ones in the time windows of 350–500 ms and 500–750 ms^15^. ERP results of another study showed that the feedback-related negativity (FRN), which peaks around 200–300 ms post-stimulus and is larger following losses than gains^16^, was significantly enhanced when another anonymous player won^17^. However, a recent study found that social comparison modulated not the FRN, but a later stage of outcome evaluation indicated by the late positive component (LPC)^18^. The conflicting results of these studies suggest that the temporal hallmarks of social comparison are not yet clear.

With EEG recording, the current study aims to investigate the temporal processing of outcome evaluation in a social comparison context. Based on prior findings^3^,^6^,^12^,^15^,^17^, we hypothesized that ERP signals would be elicited during the process of outcome evaluation and that EEG responses to different kinds of social comparing outcomes would show distinct patterns. To extend previous studies, we have revised the social comparison paradigm designed by Boksem *et al.*^17^, such that participants played the same task with two other players simultaneously. In this way, the comparison between self and others showed hierarchical characteristics; that is, a participant’s performance feedback might be different from either of other players (similar with previous studies), or be different from both of others. It would be interesting to see if participants consider the latter condition as a larger violation of the good-group-member standard (i.e., being consistent with others in the same group), and whether such an effect would manifest in electrophysiological signals.

Three ERP components, the P2, the FRN, and the LPC, were selected as neural indexes. The P2 component is associated with stimulus evaluation^19^,^20^ and attention capture modulation^21^,^22^,^23^. Information relevant to conflict with others was hypothesized to capture more attention, which would result in a larger P2 component. As mentioned above, the FRN is modulated by social contexts such that a larger interpersonal difference elicits an enhanced FRN^17^. Accordingly, we hypothesized that the FRN would increase as a function of levels of difference in social comparison. Moreover, the LPC is considered to represent levels of arousal in the late time window of approximately 500–800 ms^24^,^25^. It is larger for stimuli with high arousal levels^26^,^27^. Increased LPC amplitudes have been linked to enhanced attention^26^, more intense evaluation^24^,^25^, and better subsequent memory performance^28^,^29^. The hypothesis of this study was that the best and worst outcomes obtained in social comparison would elicit larger LPC than other conditions, since these two kinds of outcomes are more motivationally salient.

## Methods

### Participants

20 healthy students (10 females; mean age 22.65 ± 1.90 years) from Beijing Normal University participated in the experiment. All participants had normal or corrected to normal vision and had no history of psychiatric, medical, or neurological illness. All were right-handed. During debriefing, no participants self-identified as red/green color blind. All participants provided written informed consent prior to the experiment. The experimental protocol was approved by the local Ethics Committee (Beijing Normal University) and was in compliance with the ethical guidelines of the American Psychological Association.

### Procedure

To reinforce the social nature of the task, participants were told that they would play a lottery game with two other anonymous players simultaneously. In reality, there were no other people playing the game. Participants were instructed about the rules of the game and were informed that their payments depended on their choices in the task; the higher the scores participants earned the higher payment they would get at the end of the experiment. At the end of the experiment, each participant was inquired about the credibility of the cover story and none of them raised doubts.

**Figure 1:**
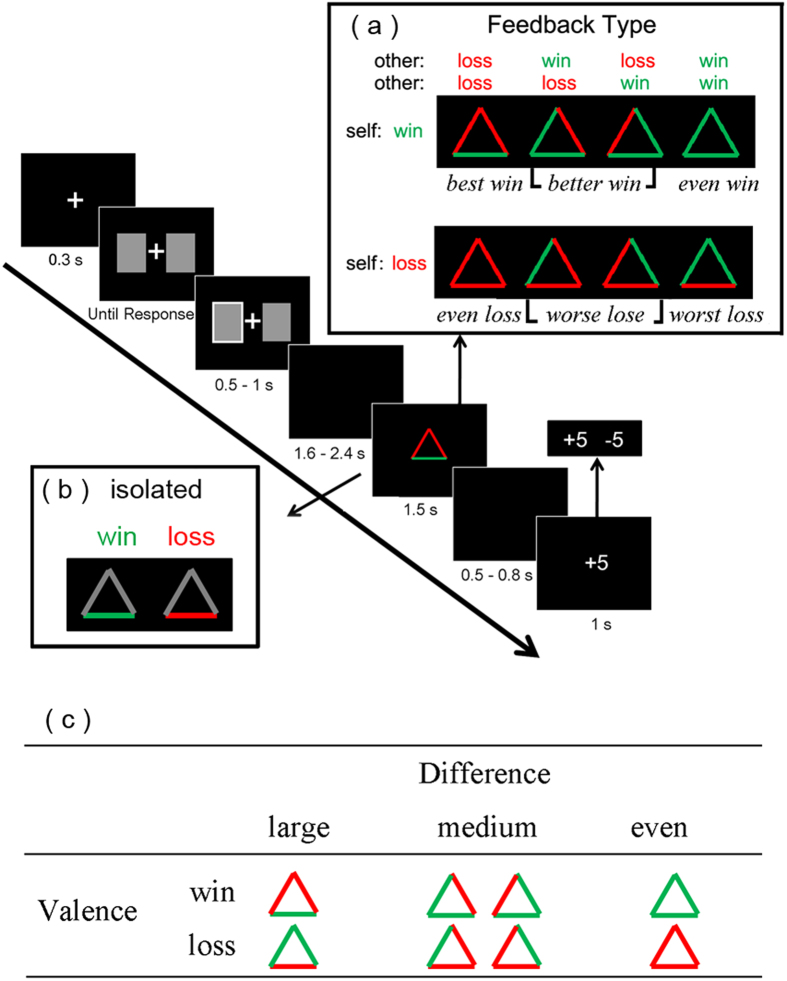
Schematic Illustration of a trial and feedback types. (**a**) Single-trial settings. A central fixation cross was presented for 0.3 s and then the participant chose between two cards. After a short delay (1.6–2.4 s), a feedback screen informed participants about all three players’ outcomes (six types in total, see upper right). Following the brief display of a blank screen (0.5–0.8 s), relevant changes in the participant’s score were displayed. (**b**) An example of outcome feedback presentation in the control block. (**c**) Six types of social feedback regarding the valence of the participant’s outcome (win/loss) and the difference between his/her outcome and the other two players’. The color of the base of the triangle represents the participant’s outcome while that of the other edges represents the other two players’. Green indicates a win whereas red indicates a loss in this example.

During the game, each participant chose one of two cards on the left and right sides of a fixation point by pressing the F or J buttons on the keyboard with his/her left or right index finger. In any given trial, one card led to win whereas the other one led to loss (see Fig. 1a). Participants were instructed that there was no relationship between the location of cards and outcomes, and that the consequence of his/her decision would not affect the consequence of other players’ choices or scores, and *vice versa*. After the participant finished his/her choice, the outcome of each trial was displayed as an equilateral triangle, with the colors of the three edges indicating the participant’s own outcome and the other two players’. The color (green/red) of each edge of the triangle represented the valence (win/loss) of each player’s outcome. The meanings of the locations and colors of each edge were counterbalanced across participants.

There were six types of feedback regarding the valence of the participant’s outcome (win/loss) and the difference between this outcome and the other two players’ (see Fig. 1c). Regarding the difference factor, there were three conditions in total: the large difference condition, in which the valence of participant’s outcome was different from that of both other players (e.g., participant wins, the other two lose); the medium difference condition, in which the valence of the participant’s outcome was the same with one of the other two players; and the even condition, in which the participant and the other two players all received the same outcome. Unbeknownst to participants, the game was preprogrammed such that different types of feedback were presented regardless of the participant’s actual choice. For win conditions, the large difference outcome, the medium difference outcome, and the even outcome were labeled as *best win* (self-win, others-both lose, 60 trials), *better win* (self-win, others-one win and one lose, 120 trials), and *even win* (self-win, others-both win, 60 trials), respectively. Likewise, for loss conditions, the large difference outcome, the medium difference outcome, and the even outcome were labeled as *worst loss* (self-lose, others-both win, 60 trials), *worse loss* (self-lose, others-one win and one lose, 120 trials), and *even loss* (self-lose, others-both lose, 60 trials), respectively.

In addition, participants also finished a control block, in which they played the same lottery game in a non-social context (i.e., isolated). This block contained two kinds of outcomes, that is, *isolated win* (60 trials) and *isolated loss* (60 trials), which were presented in a pre-determined pseudorandom order (see Fig. 1b). In this block, the outcome was also displayed as an equilateral triangle. However, only the edge that signified the participant’s own outcome would change colors (green/red) across trials, while the other two edges remained in light grey, indicating that no other people were playing with the participant. The sequence of the task block and the control block was counterbalanced across participants.

### Electrophysiological Recording and Analyses

The electroencephalogram (EEG) was recorded from 57 scalp sites using tin electrodes mounted in an elastic cap (Brain Products GmbH, Gilching, Germany) with an online reference to the left mastoid and offline algebraic rereference to the average of the left and right mastoids. Horizontal EOG was recorded from electrodes placed at the outer canthi of both eyes. Vertical EOG was recorded from electrodes placed above and below the right eye. All inter-electrode impedance was maintained at < 5 kΩ. EEG and EOG signals were amplified with a 0.01–100 Hz online band-pass filter and continuously sampled at 500 Hz/channel.

During the offline analysis, the EEG data were filtered with a 30 Hz low-pass filter (24 dB/oct) and were segmented into epochs time-locked to the onset of outcome presentation. Separate EEG epochs of 1200 ms were baseline-corrected by subtracting the average activity of that channel during the −200–0 ms baseline period from each sample. Any trial in which maximum EEG voltage exceeded a threshold of ± 100 μV during the recording epoch was excluded from further analysis. Epochs were then averaged separately for each participant and each condition.

P2 amplitudes were measured for each participant as the mean value within the 150–220 ms time window following outcome presentation. Visual detection into the averaged ERPs indicated that the P2 amplitudes were maximal at Fz along the midline electrodes. Accordingly, this electrode and five adjacent electrodes (F1, F2, FCz, FC1, and FC2) were chosen for further analysis. (The F* electrodes were the front-most row of electrodes on a 57-site cap, so there were only five electrodes around Fz.)

FRN amplitudes were measured as the mean value within the time window of 240–300 ms. Mean amplitudes of Cz, at which the mean amplitude was maximal along the midline electrodes (Fz, FCz, Cz, CPz, Pz, POz and Oz), and eight adjacent electrodes (FC1, FC2, FCz, C1, C2, CP1, CP2 and CPz) were obtained for further analysis.

LPC amplitudes were measured as the mean value within the time window of 500–800 ms. Mean amplitudes of CPz, where the LPC was maximal along the midline electrodes, and eight adjacent electrodes (C1, Cz, C2, CP1, CP2, P1, Pz and P2) were obtained for further analysis.

The P2, FRN, and LPC amplitudes were all analyzed using two-way repeated analysis of variance (ANOVA) of Valence (win vs. loss) × Difference (large difference vs. medium difference vs. even vs. isolated).

For all the analyses listed below, the significance level was set at .05. Greenhouse–Geisser corrections were used whenever appropriate. Post-hoc testing of significant main effects and interactions was conducted using the least significant difference (LSD) method. Partial eta-squared (*η*^2^_p_) values were provided to demonstrate effect size where appropriate, such that 0.05 represents a small effect, 0.10 represents a medium effect, and 0.20 represents a large effect^30^.

## Results

### Behavioral Results

The average percentage of choosing the left card was 48.4 ± 3.4%, and the percentage of choosing the right card was 51.7 ± 3.4%. The average time for decision-making was 0.77 ± 0.27 s. Because there was no optimal strategy for the participants during the task, the behavioral data were not analyzed further.

### ERP Results

#### The P2 component

**Figure 2:**
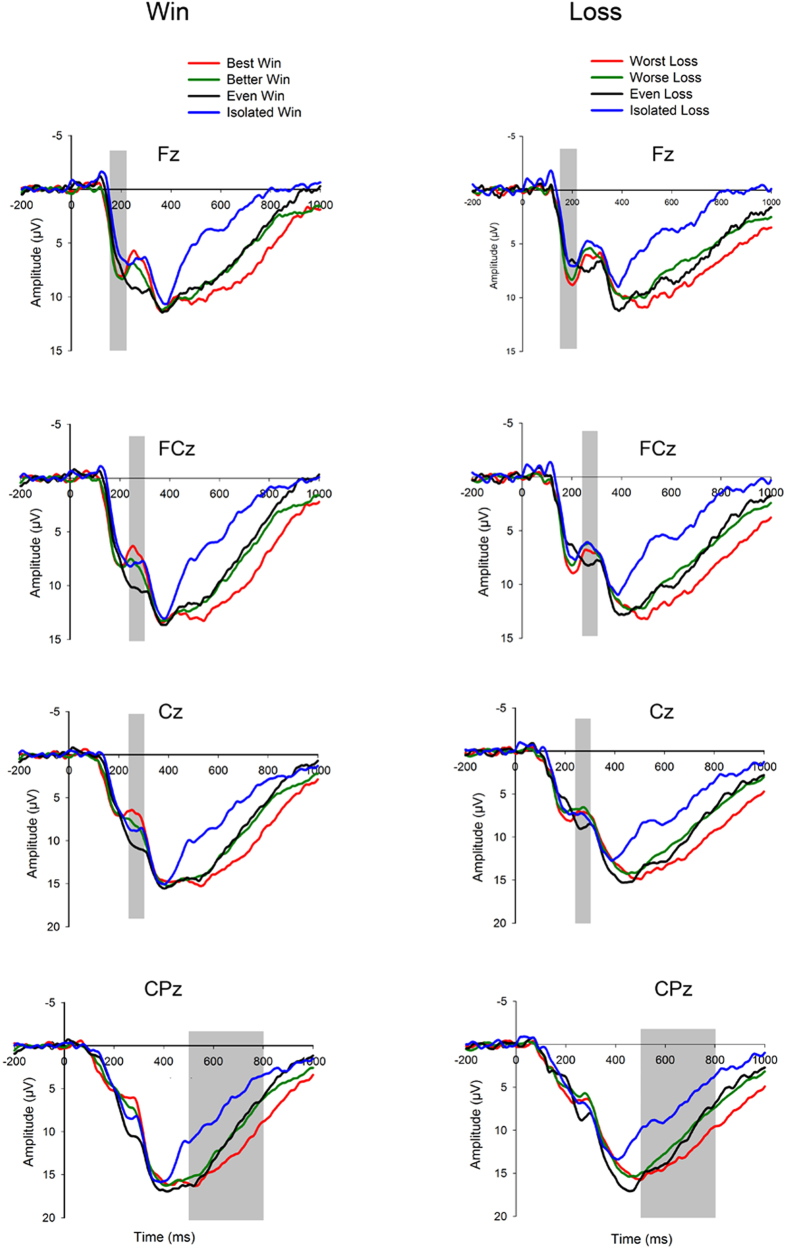
Grand-averaged ERPs at Fz, FCz, Cz and CPz for the eight types of feedback in the win and loss conditions separately. The time point 0 indicates the onset time of the outcome presentation (i.e., the equilateral triangle). Outcomes indicating differences from others elicited a larger P2 than even outcomes (upper panel). The FRN was sensitive to both value and differences from others (two middle panels). The larger difference condition evoked a larger LPC than other conditions (lower panel).

The main effect of the difference factor was significant, *F* (2,29) = 11.626, *p* = 0.001, *η*^2^_p_ = 0.380. Post-hoc analyses indicated that both the large difference (6.94 μV) and medium difference conditions (6.75 μV) elicited a larger P2 than the even condition (5.69 μV, *p* values < 0.001) and the isolated condition (4.98 μV, *p* values = 0.001). There was no significant difference between large difference and medium difference (*p* = 0.290), or between the even condition and the isolated condition (*p* = 0.179). The main effect of the valence factor was not significant, *F*(1,19) = 1.043, *p* = 0.320, *η*^2^_p_ = 0.052. No significant interaction of Valence × Difference was found, *F*(2,35) = 0.790, *p* = 0.504, *η*^2^_p_ = 0.040 (see Fig. 2).

#### The FRN component

The main effect of the valence factor was significant, *F*(1,19) = 6.171, *p* = 0.022, *η*^2^_p_ = 0.245. Losses (6.98 μV) elicited a larger FRN than wins (8.1 μV). The main effect of the difference factor was also significant, *F*(2,37) = 6.522, *p* = 0.004, *η*^2^_p_ = 0.256. Post-hoc analyses indicated that the large difference (6.62 μV), medium difference (7.03 μV), and isolated (7.43 μV) conditions all elicited larger FRN amplitudes than the even condition (9.09 μV), *p* values < 0.05. Furthermore, a significant interaction of Valence × Difference was revealed, *F*(2,37) = 4.930, *p* = 0.014, *η*^2^_p_ = 0.206. Specifically, in response to wins, the FRN amplitude increased as a function of levels of difference in social context (*best win*: 6.45 μV; *better win*: 7.75 μV; *even win*: 10.06 μV, *p* values < 0.05). The FRN amplitude evoked by *isolated win* (8.13 μV) was smaller than *best win* and larger than *even win*, but it was not significantly different from that evoked by *better win*. Regarding losses, *even loss* (8.11 μV) elicited a smaller FRN than both *worst loss* (6.78 μV), *p* = 0.028, and *worse loss* (6.30 μV), *p* = 0.001, whereas no significant difference was found between *worst* and *worse loss*, *p* = 0.183 or between *isolated loss* (6.73 μV) and other loss conditions, *p* values > 0.05 (see Fig. 2).

#### The LPC component

The main effect of the difference factor was significant, *F*(2,31) = 31.122, *p* < 0.001, *η*^2^_p_ = 0.621. Post-hoc analyses indicated that the LPC was larger in response to large difference (12.22 μV) than both medium difference (10.51 μV) and the even condition (10.66 μV), *p* values < 0.05. All of these conditions elicited a larger LPC than the isolated condition (6.64 μV, *p* values < 0.001). Neither the main effect of the valence factor, *F*(1,19) = 0.115, *p* = 0.738, *η*^2^_p_ = 0.006, nor the interaction of Valence × Difference, *F*(2,39) = 0.091, *p* = 0.965, *η*^2^_p_ = 0.005, was significant (see Fig. 2).

## Discussion

The current study had participants playing a lottery game with two anonymous players, and participants received both their own outcomes and those of the other players after each trial. We aimed to investigate the ERP correlates of social comparison outcome evaluation, which may be divided into three stages according to the results. First, the conditions indicating differences from others were discriminated from the even condition. The detection of this difference, regardless of its level, elicited a larger P2. The second stage was labeled by the FRN, which was not only sensitive to the valence of participants’ own outcomes, but also to their differences from others. Interestingly, the influence of outcome valence on the FRN showed an asymmetric pattern, with higher sensitivity to social comparison differences of wins than losses. Lastly, the large difference condition evoked a larger LPC than other conditions, suggesting an emphasis on the most salient stimuli in the third stage.

One might argue that, considering the variations of stimulus property between conditions, our ERP results may be explained in terms of perceptual difference rather than social comparison. We disagree with this viewpoint. Previous studies reveal that stimulus property (e.g., location and color) modulates early ERP components such as the C1, P1, and N1^31^,^32^, none of which are included in our analysis. The P2, FRN, and LPC components are indeed sensitive to stimulus novelty^33^,^34^, but this factor is not sufficient to explain the patterns of ERPs in the current study. Otherwise, the medium difference (240 trials in total) should elicit smaller ERPs than the larger difference and even outcome (120 trials each), which was not the case for any components in the analysis. Last but not least, the ERP patterns in the formal task significantly deviated from those when the participants played alone (i.e., the control block; see below for details). In brief, we suggest the current ERP findings are beyond perceptual difference, and should be attributed to the social context rather than perceptual processing. Below we propose our interpretation about the role of social comparison on our findings, which is divided into three stages.

### The First Stage

In the first stage, the frontal-central P2 was larger when participants faced outcomes which were different from others’. This finding suggests an early and coarse identification of distinctions between one’s own performance and others’. The P2 component may indicate the early detection of any deviation from the good-group-member standard (i.e., being consistent with others in the same group^35^), with large difference and medium difference drawing a greater amount of attention than even outcomes^23^. Additionally, the lack of sensitivity to the hierarchy of social comparison suggested that, at the early stage of outcome evaluation, social contexts which indicated people distinguishing from others might capture more attention, but detailed information was not yet processed until later stages. The P2 amplitude in the isolated condition was not significantly different from the even condition, which also suggest that at this stage, social comparison is rough and could not distinguish between the alone situation and the situation of having the same performance with others.

### The Second Stage

In the second stage, the processing of outcomes in social comparison contexts, which was coarse during the time window of the P2, turned out to be more elaborated. Consistent with classical findings^16^,^36^,^37^, monetary losses evoked a larger FRN than wins. Furthermore, the effect of social difference between the participant and other players was also significant, with a larger FRN for outcomes indicating nonconformity with others and a smaller FRN for even outcomes, regardless of outcome valence.

Disagreement with the opinions of others, nonconformity with the performance of others, and unequal incomes compared with others all induce social conflicts^2^,^3^,^15^, which trigger neural responses analogous to “reward prediction error”^36^,^37^,^38^. People expect to be consistent with others in social contexts such that the good-group-member standard is satisfied^35^. Any deviation of this expectation might be detected as a “social reward prediction error”^3^. Our results revealed that both positive and negative social reward prediction error evoked a larger FRN than even outcomes, indicating that the FRN reflects the degree of expectation violation in social comparisons, regardless of actual outcome valence^39^,^40^,^41^. We suggest this is because in social contexts, receiving a better outcome than others may elicit negative emotions from others (such as envy) and lead to disadvantaged social consequences accordingly (e.g., being isolated). In this sense, “better” outcomes may violate personal expectation as “worse” outcomes do in social comparison. Notably, the FRN in the even condition was also smaller than the isolated condition, indicating that receiving the same outcome with other people is more rewarding than the single-player context.

Interestingly, an interaction of valence by difference was found, suggesting the FRN amplitude was hierarchically sensitive to social comparison in the win condition but not in the loss condition. This asymmetry is consistent with previous findings showing a larger weight on gains than losses in social outcome evaluation^14^. The reason of this asymmetric effect is not yet clear, but it might be associated with the positivity bias in self-relevant feedback processing. Previous studies proposed that human beings evaluate information in a positive direction to achieve and maintain a positive self-concept^42^. Positivity biases documented in social cognition research suggest that reward plays a more important role than punishment in social outcome evaluation^43^,^44^, which may account for the stronger sensitivity to social comparison outcomes in response to wins than losses. When receiving monetary losses, in order to cope with potential threat to positive self-concept and the need to belong, people tend to withdraw from further attempts to evaluate detailed information and avoid upward comparisons, which could lead to a blunt social comparison evaluation^45^,^46^. Consistent with the above interpretation, the FRN following *isolated win* was significantly different from most conditions, but this is not the case for the FRN following *isolated loss.*

### The Third Stage

In the third stage, the outcomes indicating the largest social comparison difference elicited a larger LPC, which might reflect the processing of stimuli with stronger motivational relevance^47^ and higher levels of autonomic arousal^48^,^49^. In our task, both *best win* and *worst loss* indicate the participant went against the majority of the group, which might be the most rewarding/ harmful situations. In either case, timely and effective reactions would be necessary to reconcile the potential conflict between self and others in daily life. Thus, it is not surprising that these situations are evaluated as most motivational relevant, indicated by an enhanced LPC in this study. The isolated condition, irrespective of whether wins or losses, elicited the smallest LPC. This result supports our viewpoint and demonstrates that the LPC reflects an evaluation of social comparison which is insensitive to outcome valence^18^. In short, the LPC patterns indicate that participants focused on the outcomes with the most prominent social significance in the last stage of outcome evaluation, which is evolutionary adaptive and may improve the efficiency of social decision-making.

## Conclusion

Taken together, the ERP findings in the current study reveal how social comparison is processed in three stages, which requires good understanding of context-dependent social norms and social structures among group members^8^,^35^,^50^. Specifically, people are conscious of social expectations of their behaviors and promptly detect any deviation from that expectation. Thereafter, an elaborative comparison between self and others appears to be executed hierarchically, which is crucial for recognizing different kinds of social relationships. Finally, strongest motivational salience is attributed to the difference between self and the group, so as to elicit behavioral adjustment in the current social context.

To sum up, using ERP signals as temporal hallmarks, the current study demonstrates that the human brain evaluates outcomes in a social context-dependent pattern^6^,^12^,^17^. The need to belong to a social group and the associated monitoring of violations from expectancy based on the good-group-member standard may be sources of distinct neural responses to diverse social outcomes. Our findings extend knowledge about the temporal processing of social outcome evaluation by providing a temporal description of social comparison.

Finally, a few issues need to be addressed for future research. First, it would be desirable to add intermediate experimental conditions into the task design. That is, if the number of other players increases to three or more, then researchers could examine whether the ERP amplitudes would gradually change as the deviation between the participant and the majority becomes larger, or they would show an all-or-none pattern. Second, regarding that all the participants were Chinese people, we suggest follow-up researchers to recruit their samples from Western culture, which is less social comparison seeking and less sensitive to the good-group-member standard due to its individualism characteristic^51^. Replicating the current study on Western participants would help to reveal whether social comparison is susceptible to cultural variation^52^.

## Additional Information

**How to cite this article**: Luo, Y. *et al.* Social Comparison Manifests in Event-related Potentials. *Sci. Rep.* **5**, 12127; doi: 10.1038/srep12127 (2015).
